# The Relation between Frequency of E-Cigarette Use and Frequency and Intensity of Cigarette Smoking among South Korean Adolescents

**DOI:** 10.3390/ijerph14030305

**Published:** 2017-03-14

**Authors:** Jung Ah Lee, Sungkyu Lee, Hong-Jun Cho

**Affiliations:** 1 Department of Family Medicine, ASAN Medical Center, University of Ulsan College of Medicine, Seoul 05505, Korea; junga_lee@amc.seoul.kr; 2 National Evidence-Based Healthcare Collaborating Agency, Seoul 100705, Korea; wwwvince77@gmail.com

**Keywords:** electronic cigarette, e-cigarette, smoking, adolescent, frequency, tobacco

## Abstract

Introduction: The prevalence of adolescent electronic cigarette (e-cigarette) use has increased in most countries. This study aims to determine the relation between the frequency of e-cigarette use and the frequency and intensity of cigarette smoking. Additionally, the study evaluates the association between the reasons for e-cigarette use and the frequency of its use. Materials and Methods: Using the 2015 Korean Youth Risk Behavior Web-Based Survey, we included 6655 adolescents with an experience of e-cigarette use who were middle and high school students aged 13–18 years. We compared smoking experience, the frequency and intensity of cigarette smoking, and the relation between the reasons for e-cigarette uses and the frequency of e-cigarette use. Results: The prevalence of e-cigarette ever and current (past 30 days) users were 10.1% and 3.9%, respectively. Of the ever users, approximately 60% used e-cigarettes not within 1 month. On the other hand, 8.1% used e-cigarettes daily. The frequent and intensive cigarette smoking was associated with frequent e-cigarette uses. The percentage of frequent e-cigarette users (≥10 days/month) was 3.5% in adolescents who did not smoke within a month, but 28.7% among daily smokers. Additionally, it was 9.1% in smokers who smoked less than 1 cigarette/month, but 55.1% in smokers who smoked ≥20 cigarettes/day. The most common reason for e-cigarette use was curiosity (22.9%), followed by the belief that they are less harmful than conventional cigarettes (18.9%), the desire to quit smoking (13.1%), and the capacity for indoor use (10.7%). Curiosity was the most common reason among less frequent e-cigarette users; however, the desire to quit smoking and the capacity for indoor use were the most common reasons among more frequent users. Conclusions: Results showed a positive relation between frequency or intensity of conventional cigarette smoking and the frequency of e-cigarette use among Korean adolescents, and frequency of e-cigarette use differed according to the reason for the use of e-cigarettes.

## 1. Introduction 

The electronic cigarette (e-cigarette) is a battery-operated device that vaporizes a solution of nicotine, glycerol, and flavoring agents [1]. It is often advertised as a healthier alternative to conventional cigarettes or as a smoking cessation aid [1,2]. The prevalence of e-cigarette use among adolescents has been increasing in many countries [3,4,5,6,7]. Several studies have found that adolescents who used only e-cigarettes were more likely to initiate the use of combustible cigarettes later [8,9,10,11,12].

Current e-cigarette users are defined as adults and adolescents who have used them once or more during the past 30 days. It is important to distinguish established users from non-established users because there is a difference in the reason for e-cigarette initiation in addition to the extent of its use [13]. According to previous studies considering current cigarette smokers, the successful smoking cessation rate was higher among daily or intensive e-cigarette users compared with non-daily or intermittent users [14,15,16].

The general concept of current e-cigarette use is broadly defined to differentiate experimenters from regular users. Studies have argued that most adolescent e-cigarette users are experimenters and only conventional cigarette smokers tend to use e-cigarettes on a regular basis [17]. A recent study on the distribution of the frequency of e-cigarette use among adults tried to define regular users among current adult smokers and suggested more than 5 out of the past 30 days as a definition of a “persistent user” [13]. Only a few studies have reported that the frequency of e-cigarette use was particularly associated with conventional cigarette smoking among adolescents. Warner demonstrated that, even though e-cigarette use frequency rose with the amount of ever smoking in US adolescents, the frequency of e-cigarette use was not associated with the amount of conventional cigarette smoking [18].

It is important to recognize the reasons for e-cigarette initiation of adolescents because it can help us better understand what attracts adolescents to e-cigarettes. Furthermore, several reasons for initiating e-cigarettes may have increased the risk of continued use of e-cigarettes [19]. A study has reported that the reasons for e-cigarette use were related to future smoking cessation among adults [20]. Smokers who used e-cigarettes for smoking cessation showed a higher smoking cessation rate, whereas those with other reasons showed lower smoking cessation rates than non-e-cigarette users [20]. Some studies have reported predictors of continued e-cigarette use [19] and the reasons for experimentation among adolescents [21]; however, few have investigated the reasons for initiation regarding the intensity of e-cigarette use. This study aims to determine the relation between the frequency of e-cigarette use and the frequency and intensity of conventional cigarette smoking among Korean adolescents based on a nationally representative cross-sectional sample. In addition, the study identifies the association between the reasons for e-cigarette use and the frequency of its use.

## 2. Materials and Methods

### 2.1. Survey Overview and Study Participants

The Korean Youth Risk Behavior Web-Based Survey (KYRBWS) is a nationally representative cross-sectional survey of Korean middle and high school students. The KYRBWS was established in 2005 for assessing health-risk behaviors of adolescents and has provided data for the development and evaluation of school health policies and programs in Korea. The survey was approved by the institutional review board of the Korea Centers for Disease Control and Prevention (2014-06EXP-02-P-A). Written informed consent was received from all participants and their parents or legal guardians. The KYRBWS was described in detail in a previous study [22]. In brief, the KYRBWS data were collected anonymously using a multistage, stratified, cluster-sampling method. The stratification was performed on the basis of 44 provinces and types of schools according to geographic accessibility, the number of schools and population, living environment, smoking rate, and alcohol consumption. The 2015 survey included 70,362 students (ages 13–18 years) in 2400 classrooms (secondary sampling units), which included three classes considering the three year school-term (one class for each year) from each of 400 middle schools and 400 high schools (primary sampling units). Of these, 68,043 adolescents from 797 schools participated in the survey (96.7% response rate). From these participants, we analyzed 6656 adolescents who had an e-cigarette experience. Our study is based on the public use dataset (https://yhs.cdc.go.kr/new/pages/main.asp).

### 2.2. Measures

#### 2.2.1. Predictor Variables

Ever conventional cigarette smokers were defined as those who responded “yes” to the question, “Have you ever tried a cigarette, even one puff, in your life?” Among ever-smokers, current conventional smokers were defined as those who replied from “1 and 2 days” to “every day” for the question, “During the past 30 days, how many days did you smoke cigarettes, even one cigarette?” Intensity of conventional cigarette smoking was defined by the following question: “How many cigarettes did you smoke a day on average in the past 30 days?” Response options were “fewer than 1 per day,” “1 per day,” “2 to 5 per day,” “6 to 9 per day,” “10 to 19 per day,” and “20 or more per day.”

The reasons for e-cigarette use were collected from the following question: “Which of the following is your main reason for using e-cigarettes?” Response options were “it seems to be less harmful,” “for smoking cessation,” “for indoor use,” “it is easy to obtain,” “it tastes better,” “it has a good flavor,” “it does not smell like tobacco,” “curiosity,” and “other.”

We included several sociodemographic variables, such as age, gender, and school grade that might be associated with conventional cigarette smoking or e-cigarette use. 

#### 2.2.2. Outcome Variable

Ever e-cigarette use was defined by a “yes” answer to the following question: “Have you ever tried e-cigarettes?” Current e-cigarette use was defined as those who replied from “1 and 2 days” to “every day” to the question, “During the past 30 days, how many days did you use e-cigarettes?” The number of days that used e-cigarette was re-grouped into 0–2 days/month, 3–9 days/month, and ≥10 days/month. 

### 2.3. Statistical Analysis

All data were analyzed by considering both sample weights and the complex sample design of the survey. The number of participants was presented as unweighted samples of individuals who participated in the KYRBWS 2015 survey. The prevalence of demographic characteristics and e-cigarette use was presented on the basis of the weighted percentage of participants with 95% confidence intervals to represent Korean adolescents. The chi-square test was used for categorical variables. A threshold for statistical significance was set at a two-tailed *p* < 0.05 level. All data were analyzed using SPSS version 21.0 (SPSS Statistics Inc., Chicago, IL, USA).

## 3. Results

### 3.1. General Characteristics of the Study Subjects

Table 1 shows the sociodemographic characteristics and the health-risk behaviors for all participants in the 2015 KYRBWS, with 52.1% being boys and 48% being girls. The prevalence of ever conventional cigarette smokers was 17.1%, and 3.7% of participants were current daily conventional cigarette smokers. Among the participants, 9.6% had not smoked cigarettes in the last month, although they were ever conventional cigarette smokers. Additionally, 1.3% of participants had smoked conventional cigarettes only for 1–2 days within month. The prevalence of ever and current e-cigarette use was 10.1% and 3.9%, respectively. Out of the total participants, 6.0% were ever e-cigarette users but had not used e-cigarettes within a month, while 1.3% had used them for only 1–2 days per month. Daily e-cigarette users were 0.7%. 

### 3.2. Frequency of E-Cigarette Use According to Smoking Behaviors among Ever E-Cigarette Users

Out of ever e-cigarette users, approximately 60% used e-cigarettes not within 1 month. Otherwise, 16.1% used them for more than 10 days per month (Table 2). Compared with e-cigarette users for 0–2 per month, frequent users were older (16.2 years vs. 15.8 years) and were more prevalent among 12th graders than 7th graders (21.9% vs. 9.7%). Current use of e-cigarettes was more prevalent among ever conventional cigarette smokers (41.5%) than among never conventional cigarette smokers (25.3%), and e-cigarette users for more than 10/month were 2 times more prevalent among ever smokers than among never smokers (17.2% vs. 9.5%). A positive correlation was observed between the frequency of conventional cigarette smoking and the frequency of e-cigarette use. Percentage of frequent e-cigarette use was 9 times greater among daily smokers than among conventional cigarette users for <1 per month (28.7% vs. 3.5%). Smoking amount was also positively correlated to the frequency of e-cigarette use. Frequent e-cigarette use was 6 times more prevalent among smokers going for ≥20 cigarettes/day than among smokers going for <1 cigarettes/month (55.1% vs. 9.1%).

### 3.3. Reasons for E-Cigarette Use and Frequency of E-Cigarette Use

Among ever e-cigarette users, the most common reason for e-cigarette use was curiosity (22.9%), followed by the belief that they were less harmful than conventional cigarettes (18.9%), the desire to quit smoking (13.1%), and the desire to smoke indoors (10.7%) (Table 3). For infrequent e-cigarette users (<3 per month), curiosity was the most frequent reason for e-cigarette use (28.8%), whereas for more frequent e-cigarette users (>10 per month), the desire to quit smoking (21.0%) and the capacity for indoor use (19.5%) were the most frequent reasons for e-cigarette use. The belief that e-cigarettes are less harmful was a common reason for use among both less (<3 per month) and more (≥10 per month) frequent users of e-cigarettes (19.3% and 17.9%, respectively).

## 4. Discussion

This study, similar to previous studies [5,23,24], found that e-cigarette use was more prevalent among ever conventional cigarette smokers than among never conventional cigarette smokers. In addition, as the frequency and intensity of cigarette smoking increase, the percentage of frequent use of e-cigarette increases. This is different from the study conducted for U.S. adolescents, where the frequency e-cigarette use was not associated with the frequency and amount of conventional cigarette smoking [18]. This finding is plausible because even heavier adolescent smokers tend to be more nicotine-dependent [25] and would use e-cigarettes more heavily. It has been suggested that intensive or daily use of e-cigarettes, especially the tank type, may increase the chances of quitting smoking among adults [14,15]; however, no cessation aid, including e-cigarettes, has proved to be effective among adolescents [26]. Moreover, considering the potential adverse effect of nicotine on adolescent brain development [27], heavier e-cigarette use would likely cause more potential harm than benefit among adolescents.

In our study, most ever e-cigarette users were infrequent users. Even among daily smokers, more than 50% used e-cigarettes for <3 per month. This suggested that many adolescent e-cigarette users could be experimenters [17]. However, 9.5% of never smokers were frequent e-cigarette users (≥10 per month), and 3.3% were daily e-cigarette users. This means that non-smoking adolescents may be using e-cigarettes as new nicotine supplementary devices. This could be a serious issue because nicotine may delay brain development, causing problems with cognition and emotional regulation [27,28], and e-cigarette use can be a gateway to combustible tobacco use and other substance use [8,9,10]. Also a recent study reported that the frequent e-cigarette vaping increased a risk of frequent and heavy conventional cigarette smoking 6 months later [29].

Overall, the most common reason for e-cigarette use was curiosity, followed by the belief that they are less harmful than conventional cigarettes, the desire to quit smoking, and the capacity for indoor use. The reasons for use differed according to the frequency of e-cigarette use. As expected, for frequent e-cigarette users, common reasons for use were the desire to reduce/quit smoking and the capacity for indoor use. This is worrisome because adolescents who use e-cigarettes as a method to avoid an indoor smoke-free policy may lose out on the chance of quitting smoking, which can interrupt a smoke-free policy. For infrequent e-cigarette users, curiosity, better taste, and good flavor comprised half of the reasons for e-cigarette use. This group initiates e-cigarette use for recreational purposes [30], and this can increase the probability of their becoming conventional cigarette smokers or other substance users [9].

Reasons for using e-cigarettes can be important in public health implications because they influence the continued use of e-cigarettes, and nicotine use is not safe especially among adolescents [10]. A previous study showed that adolescents who first tried to use e-cigarettes because they were cheap, because they wanted nicotine, or because they wanted to quit smoking tended to continue using e-cigarettes [19]. Additionally, adults who tried e-cigarettes for goal-oriented reasons (e.g., to use e-cigarettes where smoking was not allowed or to quit) were more likely to report continued use [31,32]. In our study, many e-cigarette users replied that they used e-cigarettes for the cessation of smoking. Additionally, we found that goal-oriented use of e-cigarettes (e.g., for smoking cessation, for indoor use, or because they were less harmful, had a better taste, or had less tobacco smell) were more prevalent among frequent users of e-cigarettes (e.g., ≥3 per month). This can be a possible explanation for the association between reasons for use of e-cigarettes and the continued use of them. On the other hand, e-cigarettes can be used as a recreational device in terms of the purpose of e-cigarette uses, even in non-smoking adolescents [30]. Our findings show e-cigarettes were used as a smoking cessation aid among adolescent smokers and for recreational purposes even among non-smokers, although there is lack of evidence for their safety and efficacy in adolescents [26,27]. Therefore, we suggest the need for more education for adolescents about e-cigarettes and stricter control by the government.

Our study has several limitations. First, we could not establish any causal relations between the frequency of e-cigarette use and smoking frequency or intensity because our analysis was cross-sectional and the directionality of our findings could not be determined. Second, self-reports of e-cigarette use and smoking may differ from the actual rates. This is particularly true for smoking behavior among women, which may have been under-reported. Third, we could not evaluate why participants did not use e-cigarettes, especially among conventional cigarette only users. However, when we compared conventional cigarette only users with dual users of conventional cigarettes and e-cigarettes, dual users showed a significantly higher frequency and intensity of conventional cigarette smoking (Supplementary Table S1). Finally, we did not adjust the confounding variables to evaluate the frequency of e-cigarette use according to smoking behaviors or the reasons for e-cigarette use. Despite these limitations, however, this study has the advantage of using a large amount of nationally representative data. Our results can help in understanding the behaviors of adolescent e-cigarette users and may be usefully adopted in a future campaign to prevent adolescents from using e-cigarettes. 

## 5. Conclusions

In conclusion, the frequency of e-cigarette use was positively associated with the frequency or intensity of conventional cigarette smoking. In terms of the reasons for e-cigarette use, curiosity was the most frequent reason among less frequent e-cigarette users, whereas the desire to quit smoking and the capacity for indoor use were more frequent reasons among frequent e-cigarette users. With the rapidly increasing prevalence of e-cigarette use among adolescents, this study can further our understanding of behaviors related to e-cigarette use and aid in the appropriate control of e-cigarettes. 

## Figures and Tables

**Table 1:** Sociodemographic and smoking-related characteristics of participants (*N* = 68,043).

Characteristics		*N*	Unweighted, %	Weighted, % (SE)
Age (years)	Mean ± SE	67,671	14.96 ± 1.74	15.09 ± 0.02
Grade	7	10,786	15.9	13.7 (0.3)
	8	11,442	16.8	15.6 (0.3)
	9	12,071	17.7	17.7 (0.3)
	10	11,122	16.3	17.4 (0.3)
	11	11,113	16.3	17.6 (0.3)
	12	11,509	16.9	18.1 (0.3)
Sex	Boy	35,204	51.7	52.1 (1.4)
	Girl	32,839	48.3	47.9 (1.4)
Smoking experience	No	56,415	82.9	82.6 (0.4)
	Yes	11,628	17.1	17.4 (0.4)
Smoking within 1 month	Nonsmoker	56,415	82.9	82.6 (0.4)
	Not within 1 month	6505	9.6	9.7 (0.2)
	1–2 days/month	872	1.3	1.3 (0.1)
	3–5 days/month	403	0.6	0.6 (0.0)
	6–9 days/month	340	0.5	0.5 (0.0)
	10–19 days/month	497	0.7	0.8 (0.0)
	20–29 days/month	502	0.7	0.8 (0.0)
	Daily	2509	3.7	3.8 (0.2)
E-cig experience	No	61,387	90.2	89.9 (0.3)
	Yes	6656	9.8	10.1 (0.3)
E-cig use within 1 month	Nonuser	61,387	90.2	89.9 (0.3)
	Not within 1 month	4090	6.0	6.1 (0.2)
	1–2 days/month	873	1.3	1.3 (0.1)
	3–5 days/month	390	0.6	0.6 (0.0)
	6–9 days/month	279	0.4	0.4 (0.0)
	10–19 days/month	323	0.5	0.5 (0.0)
	20–29 days/month	196	0.3	0.3 (0.0)
	Daily	505	0.7	0.8 (0.1)

SE: Standard error.

**Table 2:** Frequency of e-cigarette use according to smoking behaviors among ever e-cigarette users of Korean adolescents (*N* = 6656).

Characteristics		Number of Days Used E-Cigarettes per Month (%, 95% CI) *			
		0–2	3–9	≥10	*p*-Value
Total	*N* (%) ^†^	4962 (73.7)	669 (10.2)	1024 (16.1)	
Age (years)	Mean (SE)	15.80 ± 0.03	15.96 ± 0.07	16.22 ± 0.06	<0.001
Grade	7	84.9 (79.1–89.3)	5.4 (3.2–9.1)	9.7 (6.3–14.6)	<0.001
	8	75.6 (71.1–79.5)	11.2 (8.6–14.6)	13.2 (10.5–16.4)	
	9	77.3 (74.3–80.1)	8.7 (6.9–10.9)	14.0 (12.0–16.3)	
	10	74.9 (71.9–77.7)	10.0 (8.4–11.9)	15.1 (12.9–17.7)	
	11	75.4 (72.8–77.8)	10.7 (9.2–12.6)	13.9 (12.0–16.0)	
	12	67.3 (63.5–70.9)	10.8 (9.0–12.9)	21.9 (19.2–24.8)	
Sex	Boys	73.5 (71.8–75.2)	10.2 (9.3–11.2)	16.3 (15.0–17.7)	0.866
	Girls	74.3 (70.9–77.4)	10.2 (8.4–12.3)	15.5 (13.0–18.4)	
Smoking experience	No	85.3 (82.5–87.7)	5.2 (3.8–7.1)	9.5 (7.6–11.9)	<0.001
	Yes	71.9 (70.2–73.5)	11.0 (10.0–12.0)	17.2 (15.9–18.5)	
Smoking within 1 month (days/month)	Nonsmoker	85.3 (82.5–87.7)	5.2 (3.8–7.1)	9.5 (7.6–11.9)	<0.001
	Not within 1 month	92.6 (91.2–93.8)	3.9 (3.0–5.0)	3.5 (2.7–4.5)	
	1–2	81.0 (76.8–84.6)	11.1 (8.4–14.7)	7.8 (5.5–11.1)	
	3–5	66.5 (59.4–72.9)	19.4 (14.6–25.3)	14.1 (9.6–20.3)	
	6–9	62.7 (55.3–69.5)	19.3 (14.2–25.8)	17.9 (13.1–24.1)	
	10–19	55.7 (50.3–61.0)	18.0 (14.0–23.0)	26.2 (21.4–31.8)	
	20–29	55.8 (49.6–61.9)	16.6 (12.8–21.4)	27.5 (22.7–33.0)	
	Daily	57.8 (55.1–60.5)	13.5 (12.0–15.1)	28.7 (26.3–31.2)	
Smoking Amount within 1 month (cigarettes/day)	None	90.4 (89.0–91.5)	4.3 (3.5–5.2)	5.4 (4.5–6.3)	<0.001
	<1	81.6 (77.2–85.3)	9.4 (6.8–12.8)	9.1 (6.5–12.6)	
	1	74.7 (68.3–80.2)	10.4 (7.2–14.8)	14.9 (10.7–20.3)	
	2–5	64.9 (61.6–68.1)	14.6 (12.5–16.9)	20.5 (18.0–23.2)	
	6–9	57.6 (54.1–61.1)	16.0 (13.9–18.5)	26.3 (23.2–29.7)	
	10–19	50.7 (45.7–55.7)	19.0 (15.8–22.8)	30.2 (26.1–34.7)	
	≥20	29.8 (23.7–36.8)	15.1 (11.2–20.1)	55.1 (48.0–62.0)	

* The percentage and confidence interval denotes column %. ^†^ The number in parenthesis denotes column %. SE: Standard error; CI: Confidence interval.

**Table 3:** Frequency of e-cigarette use according to reason for e-cigarette use among ever e-cigarette users of Korean adolescents (*N* = 6656).

Reason for E-Cigarette Use	Number of Days Used E-Cigarettes, % (95% CI) *				
	*N* (%) ^†^	0–2	3–9	≥10	*p*-Value
*N* (%) ^‡^	6656 (100)	4962 (73.7)	669 (10.2)	1024 (16.1)	
Since they might be less harmful	1254 (18.9)	19.3 (18.0–20.7)	17.9 (15.1–21.0)	17.6 (15.3–20.1)	<0.001
For smoking cession	851 (13.1)	10.6 (9.5–11.7)	18.7 (15.6–22.2)	21.0 (18.1–24.3)	
For indoor use	675 (10.7)	7.9 (7.2–8.7)	17.0 (14.0–20.3)	19.5 (17.0–22.3)	
Since they are easily obtained	94 (1.5)	0.6 (0.4–0.9)	5.2 (3.6–7.5)	3.0 (2.1–4.3)	
Since they have better taste	658 (9.6)	9.0 (8.1–10.0)	12.6 (10.2–15.6)	10.5 (8.6–12.8)	
Since they have good flavor	613 (9.3)	10.0 (9.1–10.9)	7.2 (5.4–9.7)	7.2 (5.6–9.1)	
Since they do not have the smoking smell	491 (7.5)	6.4 (5.7–7.2)	7.5 (5.5–10.2)	12.5 (10.4–15.0)	
Curiosity	1572 (22.9)	28.8 (27.3–30.4)	10.2 (7.8–13.1)	3.9 (2.9–5.4)	
Other	447 (6.6)	7.4 (6.6–8.2)	3.7 (2.4–5.6)	4.7 (3.5–6.4)	

* The percentage and confidence interval denotes row %. ^†^ The number in parenthesis denotes row %. ^‡^ The number in parenthesis denotes column %. CI: Confidence interval.

## Supplementary Materials

The following are available online at www.mdpi.com/1660-4601/14/3/305/s1. Table S1. Comparison between cigarette only users and dual users.
