# Supplementary material for: The Relation between Frequency of E-Cigarette Use and Frequency and Intensity of Cigarette Smoking among South Korean Adolescents
Source: Int J Environ Res Public Health. 2017 Mar 14;14(3):305. doi: 10.3390/ijerph14030305 (PMC5369141; doi:10.3390/ijerph14030305)
Supplement: Supplementary file 1 [file ijerph-14-00305-s001.pdf]

International Journal of
Environmental Research and Public Health

MDPI

Supplementary Materials: The Relation between Frequency of E-Cigarette Use and Frequency and Intensity of Cigarette Smoking among South Korean Adolescents

Jung Ah Lee [1], Sungkyu Lee [2] and Hong-Jun Cho [1,*]

Table S1. Comparison between cigarette only users and dual users.

		Cigarette Only User (*N* = 5856)	Dual User (*N* = 5772)	*p*-Value
		Mean±SE or N (%)		
Age (years)	Mean±SE	15.73±0.3	15.95±0.03	<0.001
Grade	7	349 (4.7)	135 (1.8)	<0.001
	8	649 (9.7)	530 (8.3)	
	9	913 (15.2)	838 (14.1)	
	10	1143 (20.4)	1208 (21.2)	
	11	1350 (24.1)	1479 (26.3)	
	12	1452 (25.9)	1582 (28.4)	
Sex	Boy	4002 (68.6)	4736 (82.7)	<0.001
	Girl	1854 (31.4)	1036 (17.3)	
Smoking within 1 month	Not within 1 month	4482 (76.7)	2023 (34.5)	<0.001
	1–2 days/month	417 (7.0)	455 (7.7)	
	3–5 days/month	170 (2.8)	233 (4.0)	
	6–9 days/month	118 (2.1)	222 (3.9)	
	10–19 days/month	143 (2.4)	354 (6.2)	
	20–29 days/month	115 (2.0)	387 (7.1)	
	Daily	411 (7.1)	2098 (36.6)	
Smoking amount within 1 month (cigarettes/day)	None	4482 (76.7)	2023 (34.5)	<0.001
	<1	428 (7.3)	410 (7.2)	
	1	179 (2.9)	266 (4.6)	
	2–5	448 (7.5)	1311 (23.2)	
	6–9	185 (3.1)	946 (16.6)	
	10–19	82 (1.5)	530 (8.6)	
	≥20	52 (0.9)	286 (5.2)	

SE: Standard error.
